# The Same but Different: Worms Reveal the Pervasiveness of Developmental System Drift

**DOI:** 10.1371/journal.pgen.1004150

**Published:** 2014-02-06

**Authors:** Eric S. Haag

**Affiliations:** Department of Biology, University of Maryland, College Park, Maryland, United States of America; University of California Davis, United States of America

For at least 50 years, biologists have appreciated that some genes are universally conserved, some are restricted to particular clades, and some are found only in one species. It would be reasonable to expect that orthologous genes persist because they retain a conserved function, and in cell and developmental genetics, “function” has been defined practically by the consequences of inactivation or inappropriate activation. This simple but powerful paradigm revolutionized many fields [e.g.], [ 1]–[4] and has produced a huge body of gene-function relationships from a few genetic model organisms. Monogenic human diseases can provide a similar sort of information about our own species when the nature of the causative mutations is known. However, the phylogenetic sparseness of research models means that even when orthologous gene perturbations have been performed, it is often difficult to know what the “same” phenotype even means.

The ideal approach to characterizing the conservation of gene function would compare the effects of perturbing ortholog activity in organisms with similar anatomy, physiology, and laboratory tractability. Though we might expect that under these circumstances essentially all ortholog perturbations would give identical results, a small but growing literature suggests this is not necessarily the case. For example, a survey of over 100 transcription factor knockouts in *Candida albicans*, a distant relative of *Saccharomyces cerevisiae*, allowed the phenotypes of orthologs to be compared [5]. Most were similar, but a small number of striking shifts in the “wiring” of otherwise conserved metabolic circuits were observed (one previously described [6]). Similarly, both *fushi tarazu* and *oskar*, first identified for their roles in patterning the *Drosophila melangaster* embryo, may have initially functioned in the central nervous system and were only later co-opted into early development [7], [8]. These studies confirm that even when a trait is under strong stabilizing selection, with enough time, the orthologous genes that produce it can evolve surprisingly distinct roles—a phenomenon called developmental system drift (DSD) [9]. In a recent study in *PLOS Genetics*, Verster and colleagues [10] have undertaken a systematic search for DSD in more closely related animals.

The experimental system used by Verster et al. [10], *Caenorhabditis* nematodes, is especially advantageous for large-scale comparisons of gene function. Across the over 20 easily cultured species in the genus, anatomy and the cell lineages that produce it are essentially invariant, so there is no doubt of homology [11], [12]. RNAi knockdown is broadly applicable, though only a few species are susceptible to the simplest, food-borne method of introducing dsRNA [13]–[16]. For self-fertile species, such as *C. elegans* [2] and *C. briggsae* [17]–[19], screens for recessive mutations impacting development are also simple to conduct. These approaches have shown that, as expected, many genes do have highly similar knockdown/knockout phenotypes. However, surprising functional divergence has also been reported for orthologs regulating processes as diverse as sex determination [20]–[22], early embryonic patterning [23], vulval development [19], [24], and excretory physiology [25]. These differences indicate that even organs that are identical at the cellular level across a range of species can experience rapid DSD. However, these cases were not necessarily representative of the genome as a whole, and for genes involved in sex determination interpretation is complicated by the convergent evolution of hermaphroditism in *C. elegans*, *C. briggsae*, and *C.* sp. 11 [26], [27].

To give an unbiased estimate of the extent of DSD, Verster et al. [10] performed what may be the largest comparative analysis of gene function ever. Starting with over 1,300 genes both with strong RNAi phenotypes in *C. elegans* and with *C. briggsae* orthologs, they systematically compared the effects of knockdown in both species. After imposing several control filters, they found 91 cases of likely functional divergence (as defined by qualitatively different phenotypes), or about 7%. Though some of these may be false positives due to differential knockdown efficacy, careful quantitation for a sample of genes suggests that the fraction attributable to such artifacts is quite small. The set of genes with different phenotypes includes a disproportionately large number of transcription factors and of genes restricted to the nematode phylum, but few genes related to universal processes, such as protein synthesis factors. This makes some sense—knockdown of genes essential to cell viability will generally be lethal across the board—while transcription factors and new genes are likely to have more restricted roles and thus more potential for deviation.

**Figure 1:**
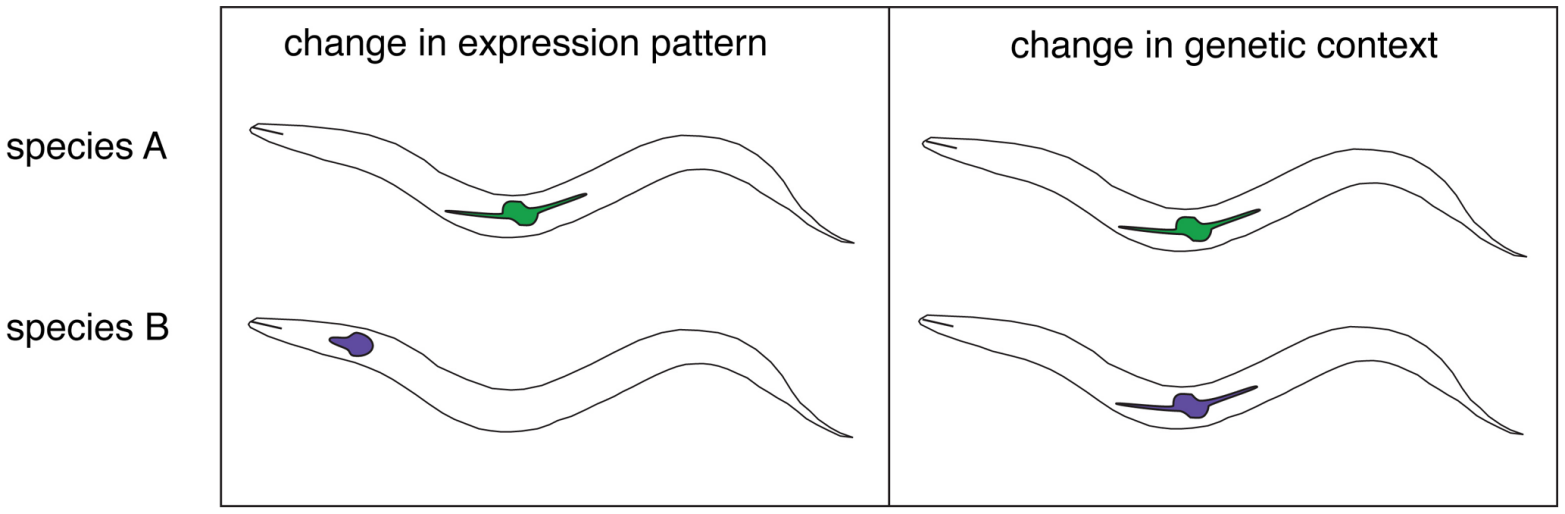
Two mechanisms for the functional divergence of orthologous genes in *Caenorhabiditis*. Left: The site of expression of orthologous genes (depicted by colored shapes) has diverged in Species A and Species B and is essential for the normal development and/or function of their respective tissues. A promoter swap is necessary and sufficient for cross-species rescue of a null mutation. Right: The expression of the orthologs has not changed, yet the consequence of inactivation produces a distinct phenotype in each. Cross-species rescue is effective without alteration of the ortholog, indicating that the locus of functional evolution is not the gene itself, but in the context for the gene's activity.

The above quantification of DSD is a major contribution, but Verster et al. went further to explain *how* divergent knockdown phenotypes evolved. They considered three possibilities, including (1) a change in expression pattern, (2) changes in protein sequence that alter the molecular function, and (3) changes in interacting genes that alter the role or required expression level of orthologs. These were then tested using reporter constructs (to infer expression patterns) and gene chimeras that mixed and matched the regulatory and coding sequences from the two species. For some orthologs, different expression patterns were observed and use of the endogenous promoter was required for cross-species rescue. This supports the first hypothesis above. Evidence for the third hypothesis was found as well. If a contextual change alone is responsible for the distinct phenotypes, then the genes themselves should be interchangeable between species. Indeed, several cases were found in which orthologs with distinct phenotypes exhibited complete cross-species transgene rescue. Recent studies of the Lef/TCF homolog *pop-1* [23] and of genes regulating germline sex determination [20], [21] and vulval development [28] have also revealed important roles for genetic context in determining the outcome of ortholog inactivation. Taken together, it appears that gene regulatory networks are constantly being reconfigured even when phenotypes are not (Figure 1), the essence of DSD.

Interestingly, in six attempts, Verster et al. failed to identify a clear case where genes with different knockdown phenotypes required the conspecific coding sequence to rescue a mutant. This is a small set of data, but the result is interesting given the different phenotypes and the substantial amino acid divergence between orthologs tested. This suggests that the main engines of DSD may be ongoing fluctuations in the regulation of gene expression and the shifting molecular context that such regulatory changes impart in a given cell type. For example, a novel expression domain for a transcriptional regulator may make another such regulator partially or completely redundant, with the excess capacity now capable of being shifted to either restore conservation (by a reversal) or to create DSD [29]. The need to simultaneously accommodate directional selection in one aspect of a pleiotropic regulator while retaining function of another under strong purifying selection may further accelerate DSD [30]. It will thus be of great interest to determine whether there is a correlation between a gene's tendency to exhibit DSD and the extent of its pleiotropy.

Few animals are as amenable to reverse genetics as *Caenorhabditis* nematodes, the study of which has now created an important insight into the divergence of gene function among close relatives. However, the recent application of custom site-specific nucleases [e.g., 31] promises to greatly accelerate this kind of work in a range of systems. The main constraint seems to be the ability to introduce the effectors into a single-cell stage of development (i.e., an egg or zygote) and raise the progeny long enough to identify new mutants. This may exclude many species, but it opens up hundreds or thousands to rigorous genetic manipulation for the first time. It will be exciting to see the results of these studies in the near future.
